# Regulation of tumor angiogenesis by the crosstalk between innate immunity and endothelial cells

**DOI:** 10.3389/fonc.2023.1171794

**Published:** 2023-05-10

**Authors:** Svenja Ebeling, Anita Kowalczyk, Diego Perez-Vazquez, Irene Mattiola

**Affiliations:** ^1^ Institute of Microbiology, Infectious Diseases and Immunology (I-MIDI), Charité - Universitätsmedizin Berlin, Corporate Member of Freie Universität Berlin, Humboldt-Universität zu Berlin, and the Berlin Institute of Health, Berlin, Germany; ^2^ Laboratory of Mucosal and Developmental Immunology, Deutsches Rheuma-Forschungszentrum (DRFZ), an Institute of the Leibniz Association, Berlin, Germany

**Keywords:** myeloid cells, innate lymphocytes, endothelial cells, cancer, angiogenesis

## Abstract

Endothelial cells and immune cells are major regulators of cancer progression and prognosis. Endothelial cell proliferation and angiogenesis are required for providing nutrients and oxygen to the nascent tumor and infiltration of immune cells to the tumor is dependent on endothelial cell activation. Myeloid cells and innate lymphocytes have an important role in shaping the tumor microenvironment by crosstalking with cancer cells and structural cells, including endothelial cells. Innate immune cells can modulate the activation and functions of tumor endothelial cells, and, in turn, endothelial cell expression of adhesion molecules can affect immune cell extravasation. However, the mechanisms underlying this bidirectional crosstalk are not fully understood. In this review, we will provide an overview of the current knowledge on the pathways regulating the crosstalk between innate immune cells and endothelial cells during tumor progression and discuss their potential contribution to the development of novel anti-tumor therapeutic approaches.

## 1 Introduction

The endothelium consists of a monolayer of endothelial cells that lines the inner surface of blood and lymphatic vessels (1). Endothelial cells regulate the function of the circulatory system, such as the blood flow and the blood pressure, as well as vessel permeability and coagulation (1). Blood vessels are formed during embryonic development, in a process called vasculogenesis, and infiltrate every tissue of the organism to provide oxygen and nutrients required for cell survival (1, 2). The mature endothelium is composed of a layer of tightly joint endothelial cells surrounded by a basal muscular layer that regulates vasoconstriction and vasodilation (1). In homeostatic conditions, endothelial cells are in a quiescent state characterized by minimal cell proliferation, and are constantly subjected to autocrine, endocrine, and paracrine signals. Pathways regulated by Wnt, Fibroblast growth factor 2 (FGF-2), Bone Morphogenetic Protein 9 (BMP-9), and Angiopoietin 1 (Ang-1) are required for the maintenance of endothelial cell identity and physical properties, as for preventing the alteration of the blood flow, the vascular permeability and the delivery of nutrients and oxygen (3). Although vascular endothelial growth factor (VEGF) is critical for developmental and pathological angiogenesis, recent evidence showed that autocrine VEGF could also contribute to the maintenance of vascular homeostasis (4).

Beyond its importance for nutrient and oxygen transport, the endothelium is central for the recruitment of immune cells to inflamed tissues (5, 6). Acute inflammation involves the rapid recruitment of leukocytes and requires endothelial cell activation, namely the acquisition of new capacities by resting endothelial cells (6). Endothelial cell activation is characterized by two types of response: a rapid response that is independent of new gene expression, called type I activation, and a slower response that is dependent on new gene expression, called type II activation (6). The latter is triggered by mediators released from activated leukocytes, such as tumor necrosis factor (TNF), interleukin 1 beta (IL-1β) and 6 (IL-6), which induce endothelial cell acquisition of a pro-inflammatory transcriptional program (6, 7). Activated endothelial cells produce Nitric Oxide (NO) that promotes vasodilation and further facilitates immune cell trafficking (8). Upon activation, endothelial cells upregulate the expression of adhesion molecules, such as E-selectin and P-selectin, intercellular adhesion molecule 1 and 2 (ICAM-1 and 2), vascular cell adhesion molecule 1 (VCAM-1), and integrins, and downregulate other intercellular adhesion molecules, such as VE-Cadherin, to enhance leukocyte migration across the endothelial cell layer (9). The process begins with the formation of transient adhesive interactions between free-flowing leukocytes and activated endothelial cells. Chemokines (CC−chemokine ligand 2 (CCL2) and CXC−chemokine ligand 10 (CXCL10)) and pro-inflammatory mediators (TNF and IL-6) secreted either from the activated endothelium (10) or from surrounding immune cells further increase leukocyte adhesion and promote crawling over the luminal endothelial surface (9). The transendothelial migration of immune cells mainly occurs by paracellular diapedesis, with leukocytes passing through the endothelial cell layer where the intercellular contacts are loosening (11). Only in a minority of cases, the migration of leukocytes occurs *via* transcellular diapedesis, characterized by intraluminal crawling of leukocytes (12), eventually when endothelial cell junctions are too tight (13). Similar receptors and adhesion molecules are involved in both types of diapedeses (14), but the molecular mechanisms that decide the use of one type over the other are still not fully understood. Excessive exposure of endothelial cells to pro-inflammatory mediators, such as TNF and interferon γ (IFN-γ) (15, 16), or bacteria (17) leads to endothelial cell apoptosis and perturbation of vascular permeability. IL-10, an anti-inflammatory cytokine largely described to promote resolution of inflammation and prevent tissue damage (18), was shown to prevent such endothelial cell apoptosis (17), to preserve the expression of endothelial junction proteins, namely occludin and claudin 5 (16), and to maintain proper vascular permeability during inflammation (15).

Depending on the surrounding environment, endothelial cells can acquire different phenotypes and exert different functions. This concept was extended when highly powered single-cell (sc) transcriptomics studies allowed a more detailed characterization of endothelial cell heterogeneity in homeostasis and disease. scRNA sequencing of lung peritumoral and tumoral endothelial cells identified endothelial cell populations endowed with diverse functions correlated with different grades of activation (19). Human pulmonary arterial endothelial cells expressed genes implicated in vascular integrity and homeostasis, whereas postcapillary venous were characterized by the expression of genes involved in leukocyte recruitment, tissue perfusion and blood pressure (19). Interestingly, peritumoral capillary endothelial cells expressed genes associated with semi-professional antigen presentation, including *HLA*, but not *CD80* and *CD86*, and with scavenging activity, such as *MARCO* (19). This suggested a role for peritumoral and tumoral endothelial cells in tumor immune surveillance (19). The capability of the endothelium to adapt to changes in the microenvironment highlights its central role in the regulation of tissue homeostasis and points it as a target for novel therapeutic approaches to control inflammation.

The endothelium not only responds to inflammatory signals but constantly communicates with the surrounding cells to provide the required oxygen, nutrients, and macromolecules to every organ in the body (1). Angiogenesis, namely the formation of new vessels from existing ones aimed to provide further nutrients and oxygen, is required from the onset of tumors (1-2 mm of tumor size) (20), and it is widely recognized as an essential hallmark of cancer (21). Tumor cells promote an angiogenic switch in endothelial cells by secreting pro-angiogenic factors such as VEGF, platelet-derived growth factor (PDGF), and angiopoietin 2 (Ang-2), inducing the destabilization of the endothelial cell barrier and promoting endothelial cell migration and proliferation (22). As tumor growth progresses, the high proliferation of cancer cells demands a supply of oxygen (and nutrients) that cannot be satisfied by the existing vasculature (23). Therefore, cancer cells express hypoxia-inducible factor 1 (HIF-1) and secrete VEGF to promote angiogenesis (24). The newly formed vessels within the tumor have an immature phenotype characterized by limited contact between endothelial cells and defective or discontinuous basement membrane. This prevents tumor endothelial cells from properly responding to physiological stimuli (25). Tumor endothelial cells are characterized by a pro-angiogenic phenotype, with high expression of VEGF receptors (VEGFR1 and R2), which pathways support endothelial cell survival and proliferation, and release of matrix metalloproteinases (MMP), involved in the remodeling of the extracellular matrix (ECM) and thus facilitating endothelial cell migration (26, 27). Tumor endothelial cells also show reduced expression of leukocyte binding molecules, including ICAM-1 and 2, VCAM-1, E-selectin and CD34, leading to reduced response to inflammatory signals and defective recruitment of immune cells (28, 29). Moreover, the structural and transcriptional abnormalities in the tumor endothelium facilitate the extravasation of cancer cells and promote metastasis development (30). Altogether, these phenotypical changes in tumor endothelial cells limit immune cell activation and infiltration in the tumor microenvironment (TME), favoring cancer progression. Therefore, a better understanding of the mechanisms regulating endothelial cell manipulation in cancer may improve the efficacy and success of current immunotherapeutic approaches.

Immune cells are part of the TME and have a key role in regulating cancer progression (21). The immune system comprises two branches, consisting of innate and adaptive immunity. Adaptive immune cells are endowed with memory functions and are involved in long-lasting immune responses (31). Conversely, innate immune cells represent the first line of immune defense and promptly respond to environmental challenges mounting appropriate immune responses and activating adaptive immunity (31). Innate immune cells, including myeloid and lymphoid cells, control the early stages of tumor progression by directly interacting with tumor cells and by shaping the TME (31), in a process called cancer immunoediting (21). However, innate immune cell recruitment and activation in the TME is strictly dependent on endothelial cells, which engage a bidirectional crosstalk with innate immune cells (7). The interaction between endothelial cells and innate immune cells represents an important cellular circuit in the regulation of cancer progression. Here, we will provide an overview of the cellular pathways by which myeloid cells and innate lymphocytes interact with endothelial cells in the TME and discuss potential implications of such interactions for anti-cancer treatments.

## 2 Myeloid cells and angiogenesis in cancer

**Figure 1:**
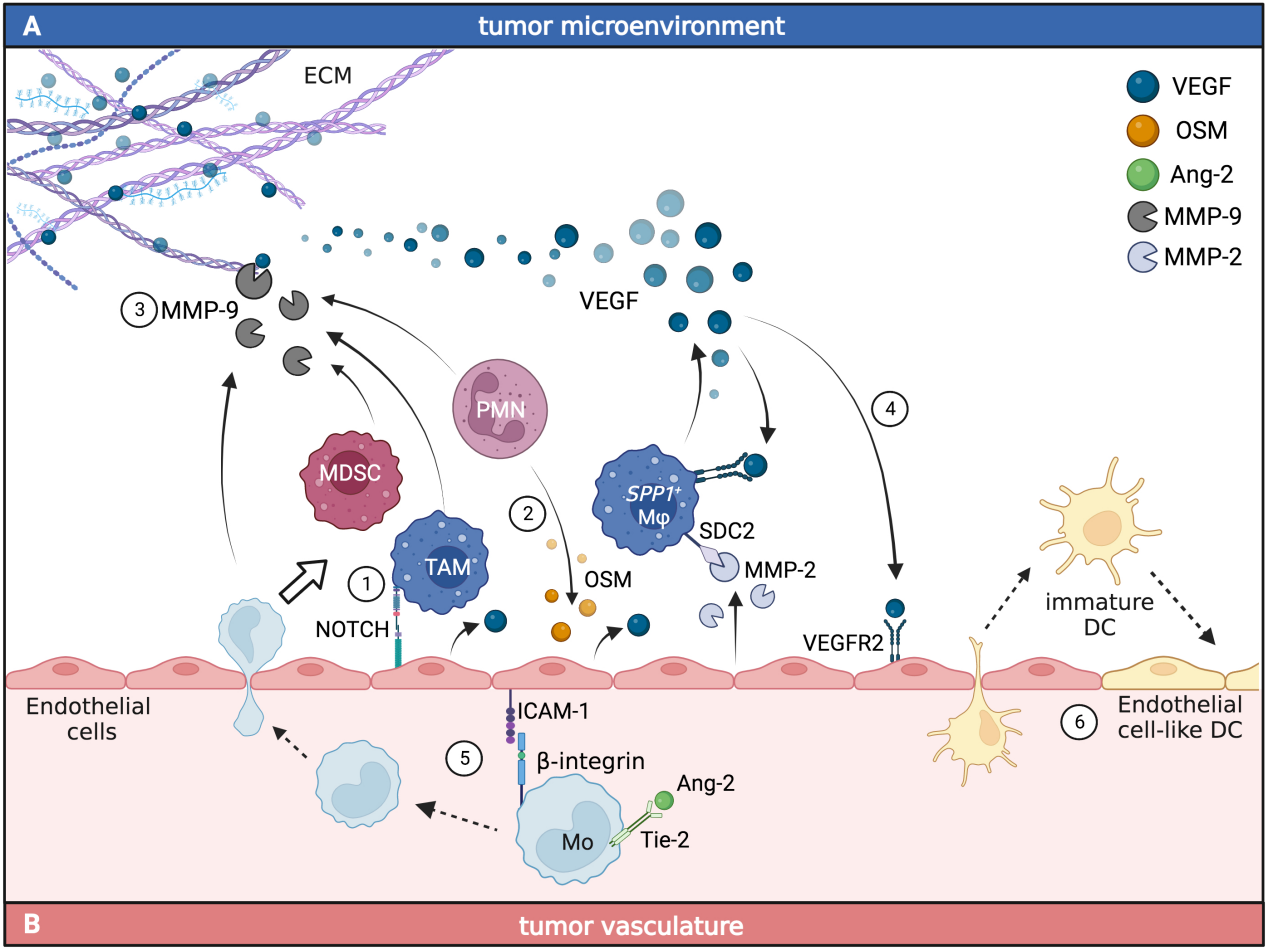
Myeloid and endothelial cell crosstalk promotes angiogenesis in the TME. The interaction between endothelial cells and tumor-infiltrating myeloid cells **(A)** or circulating myeloid cells **(B)** leads to the accumulation of pro-angiogenic signals in cancer. In the TME **(A)**, myeloid cells can engage direct or indirect crosstalk with endothelial cells. TAM and polymorphonuclear cells (PMN) can directly induce the production of VEGF in endothelial cells through the activation of Notch1 (1) and OSM-dependent (2) pathways. Together with MDSC, TAM can also indirectly induce the release of VEGF from the ECM through the production of MMP-9 (3). VEGF production from TAM activates VEGFR2 signaling in endothelial cells (4), promoting angiogenesis and leading to increased tumor vascularization. In the tumor vasculature **(B)**, monocytes expressing Tie-2 respond to Ang-2 by increasing the expression of β-integrins that bind ICAM-1 on endothelial cells facilitating monocyte migration to the TME (5), where they differentiate into tumor-associated myeloid cells. Circulating immature DC can support tumor neovasculogenesis by adopting endothelial cell-like functions and being incorporated into newly forming vessels (6). Figure generated with BioRender.

Myeloid cells are circulating and tissue-resident innate immune cells having an important role in sensing the environment, patrolling tissues, and orchestrating immune responses (32). Myeloid cells constantly crosstalk with non-hematopoietic cells composing tissue niches and with innate and adaptive lymphocytes, promoting their recruitment to sites of inflammation and their activation (33). Research done over two decades provided evidence of myeloid cells being critical players in cancer, by either favoring tumor progression or regression, depending on the regulatory features exerted in the TME (34–36). In the next session, we will give an overview on the pathways regulating the crosstalk between the different subsets of myeloid cells, namely macrophages, neutrophils, dendritic cells and monocytes, and endothelial cells in cancer, with a particular focus on intestinal tumors (**Figure 1**).

### 2.1 Tumor-associated macrophages and endothelial cell crosstalk

Tumor-associated macrophages (TAM) are one of the most represented populations of myeloid cells in the TME (36, 37). The initial definition of TAM subsets based on M1 (pro-inflammatory and tumor suppressing) and M2 (anti-inflammatory and promoting tumor progression) (35, 38, 39) was recently overcome by a more heterogenous definition based on transcriptional programs representative of different phenotypes and functions, including activation and cytokine production (40–43). Interestingly, TAM were shown to secrete several pro-angiogenic cytokines and growth factors, such as VEGF (44–46), MMP (46–48), IL-10 (40, 46, 47, 49, 50), IL-8 (45, 46), FGF2 (45, 51, 52) and placental growth factor (PlGF) (52), allowing them to modulate angiogenic pathways.

In relation to VEGF regulation and signaling, macrophages were shown to play a dual role. On the one hand, Stefater and colleagues showed that, during development, the stimulation of non-canonical Wnt-signaling (Wnt11 and Wnt5A) induced retinal CD11b^+^ F4/80^+^ macrophages to secrete soluble VEGFR1, sequestering VEGF from the surrounding environment and thus reducing its availability for endothelial cells (53). This prevented uncontrolled outgrow of vessels and suggested a crucial role of macrophages for proper growth of organized vessels (53). On the other hand, in cancer, several studies linked macrophages to increased levels of VEGF in the TME. Hypoxia (54) and HIF-1α-mediated signaling (48) could induce the production of VEGF in TAM, suggesting that they could be a source of VEGF in the TME and could promote tumor angiogenesis. TAM also express VEGFR1 and VEGFR2 (55, 56) having the potential to directly bind and respond to autocrine or paracrine VEGF signaling in the TME. The inhibition of the VEGF/VEGFR2 axis in TAM led to reduced secretion of VEGF, suggesting that TAM could contribute to the accumulation of VEGF in the TME upon activation of VEGFR2 pathways (57). Interestingly, the VEGF expression profile in TAM correlated with the one of MMP-9, a matrix metallopeptidase contributing to the degradation of the ECM and facilitating endothelial cell migration (58). In mouse models of glioblastoma, MMP-9 secreted by F4/80^+^ macrophages triggered the local activation of sequestered VEGF bound to the ECM, making it available for binding to VEGFR2 and leading to endothelial cell activation (48). In a mouse model of breast cancer (PyMT), the depletion of intratumoral macrophages (colony stimulating factor 1 (CSF-1) null mutant mice) or the conditional knock-out of their secreted VEGF led to a delay of the angiogenic switch of the tumor vasculature (59–61). Altogether, these findings argue for an active role of TAM in promoting tumor angiogenesis through VEGF- and MMP-9-dependent pathways.

The recent development of next-generation sequencing techniques combined with retrospective studies on cancer patients opened new possibilities to better characterize the macrophage subsets in cancer and to further define the pathways involved in their angiogenic signaling. Regarding intestinal cancer, it was thereby possible to identify different macrophage signatures that could correlate with either good or bad prognosis (62). For instance, in colorectal cancer (CRC) patients, high infiltration of CD163^+^ TAM was linked to poor outcome (63, 64), whereas infiltration of CD206^+^ TAM correlated with better prognosis (65, 66). Notably, both markers were originally linked to a M2-like profile and therefore a tumor-promoting phenotype (67). Further studies suggested that macrophage transcriptional landscapes in different types of cancer were influenced by the cytokine signature of the respective TME (68). In human CRC, a subset of TAM characterized by SPP1 gene expression was highly enriched in cancerous tissue while being largely absent from healthy tissue (69). Notably, *SPP1^+^* TAM showed an increased expression of VEGF and Syndecan-2 (SDC2) (69), a transmembrane proteoglycan that binds MMP-2, a metalloprotease released by endothelial cells upon hypoxic conditions (70). MMP-2 is involved in the degradation of the ECM and thereby favors the formation of the tumor vasculature (71). These findings suggest that the crosstalk between *SPP1^+^* TAM and tumor endothelial cells promotes tumor angiogenesis. A recent study in mouse and human hepatocellular carcinoma identified a population of TAM and a population of tumor endothelial cells, FOLR2^+^ macrophages and PLVAP^+^ endothelial cells respectively, that resembled fetal characteristics (72). Both cell subsets were involved in implementing the onco-fetal features of the TME by interacting *via* Notch and VEGF signaling and contributing to the formation of an immunosuppressive environment (72). However, further investigations are required to assess a potential crosstalk of such fetal-like TAM and endothelial cell populations in the TME.

Tie-2-expressing macrophages (TEM) represent a macrophage subset characterized by high pro-angiogenic features. Tie-2 is the tyrosine kinase receptor for Ang-1 and Ang-2, molecules that regulate angiogenesis and endothelial cell survival, proliferation and migration (73). TEM were described to be one of the major drivers of angiogenesis in a mouse model of spontaneous pancreatic cancer (RIP-Tag2) and orthotopic brain tumors (74). TEM infiltrated several human cancers (75), and promoted tumor growth and metastases in mouse models of breast (PyMT) and pancreatic (RIP-Tag2) cancer by favoring angiogenesis (76). Notably, Ang-2 induced the upregulation of β_2_-integrin on monocytes, which interacted with ICAM-1 expressed by endothelial cells and thereby facilitated the migration of monocytes, leading to accumulation of monocyte-derived macrophages in the TME (77). Conversely, in homeostatic conditions, Ang-2-induced Wnt7B production in macrophages promoted apoptosis of endothelial cells, leading to restrained vascular growth (78–80). Thereby, depending on the tissue environment, Ang-2 potentially induces different regulatory pathways in macrophages and has a dual role in the macrophage-endothelial cell crosstalk. However, it remains elusive whether a cancerous environment could trigger either pathway.

Taken together, these findings suggest that a better understanding of TAM and TAM-endothelial cell interactions is required to identify novel pathways and disclose putative targets for innovative therapeutic approaches aimed at restraining tumor angiogenesis.

### 2.2 Angiogenic features of circulating myeloid cells

In addition to macrophages, other cells of the innate immune compartment such as neutrophils and myeloid-derived suppressor cells (MDSC) regulate angiogenesis through similar signaling pathways. Kujawski and colleagues reported that upon STAT-3-activation MDSC showed pro-angiogenic features mediated by the secretion of VEGF and MMP-9 (81). Interestingly, in a mouse model of spontaneous cervical cancer (K14-HPV/E(2) mice), MMP-9^+^ neutrophils represented an alternative source to TAM in providing MMP-9 (82) and, in murine models of colorectal cancer (MC26) and lung carcinoma (3LL), MDSC-derived MMP-9 was associated to enhanced tumor vascularization (83). In addition, when co-cultured with human breast cancer cells, neutrophils produced high levels of oncostatin M (OSM) (84), which was shown to act on the endothelium *via* STAT-3/VEGF signaling *in vivo* and to enable the remodeling of the TME by activating cancer-associated fibroblasts (CAF) (85, 86). In several murine transplantable models of cancer, MDSC were found to be the predominant source of prokineticin-2 (Bv8), a secreted protein involved in tissue-specific angiogenesis, which promoted vessel formation and tumor growth (87). Interestingly, the migration of these Bv8^+^ MDSC from the bone marrow into the peripheral blood and TME was mediated by endothelial cell-derived granulocyte colony stimulation factor (G-CSF) (87). This suggested for a bidirectional crosstalk of circulating myeloid cells and endothelial cells in cancer.

Conversely to neutrophils and MDSC, dendritic cells (DC) were described to rather have anti-angiogenic and tumor-suppressing features (38, 88, 89). Conventional DC suppressed tumor angiogenesis through the secretion of pro-inflammatory cytokines and chemokines, including IL-12, IL-18 and CXCL9 (89, 90). In a xenograft model of ovarian carcinoma, high infiltration of plasmacytoid (p)DC and low infiltration of myeloid-derived (m)DC was observed (90). Notably, only mDC were shown to produce IL-12 and to exert anti-angiogenic functions in the TME, whereas pDC were shown to interact with the vasculature by secreting IL-8 and IFN-α (90). In a xenograft model of breast cancer, IFN-α inhibited endothelial cell proliferation and thus impaired tumor vasculature formation (91). Thereby, the TME favored the infiltration of angiogenic subsets of DC and suppressed the anti-angiogenic functions of mDC. In a mouse model of ovarian cancer, immature DC (defined as CD11c^+^ DEC-205^+^ CD8α^+^ CD80^-^) were also found in the TME, where they were recruited in a β-defensin-dependent manner (92). Those tumor-associated immature DC co-localized with endothelial cells to form new vascular structures and promote neovasculogenesis (92). Therefore, depending on the origin and the differentiation stage, DC could exert either pro- or anti-angiogenic functions.

Monocytes are circulating cells that once recruited to the TME can differentiate into DC, MDSC or macrophages (93), depending on the cytokine and growth factor signature of the TME (94–97). However, monocytes could also contribute to tumor angiogenesis before differentiating into tumor-associated myeloid cell subsets (34, 47). For instance, in a xenograft model of colorectal cancer, a pro-angiogenic monocyte subset was identified, defined by the expression of both CD14 and CD16 and the secretion of MMP-9, which exerted tumor-promoting functions (98). These pro-angiogenic monocytes also expressed Tie-2 and extravasated into the TME in a VEGF-dependent manner (98). In general, CD11b^+^ myeloid cells, including monocytes, were shown to migrate towards and differentiate upon tumor-specific gradients and factors such as HIF-1α (48) or Ang-2 (47, 74, 76). Nevertheless, due to the heterogeneity of monocyte differentiation stages and subsets, the understanding of the mechanisms by which *bona fide* monocytes directly interact with endothelial cells in cancer needs further investigation.

## 3 Innate lymphoid cells and tumor vasculature

Innate lymphoid cells (ILC) are innate lymphocytes residing at barrier tissues that are endowed with immunomodulatory features and have an important role in maintaining tissue homeostasis (99). Based on the expression of master regulator transcription factors and the secretion of peculiar cytokines, ILC can be discerned into three main groups, namely group 1 ILC (ILC1), group 2 ILC (ILC2), and group 3 ILC (ILC3) (100). ILC1 express T-bet and are characterized by the secretion of pro-inflammatory cytokines, including IFN-γ; ILC2 express GATA-3 and secrete type 2 cytokines, including IL-5 and IL-13; and ILC3 express RORγt and are characterized by IL-17 and IL-22 production (99, 100). Due to their cytokine-dependent immunomodulatory features, ILC1, ILC2, and ILC3 are defined as helper-like ILC (101). NK cells represent the first discovered population of ILC (102) and consist of circulating innate lymphocytes characterized by T-bet and Eomes expression and are endowed with cytotoxic functions (102). Therefore, NK cells are defined as cytotoxic ILC (101).

**Figure 2:**
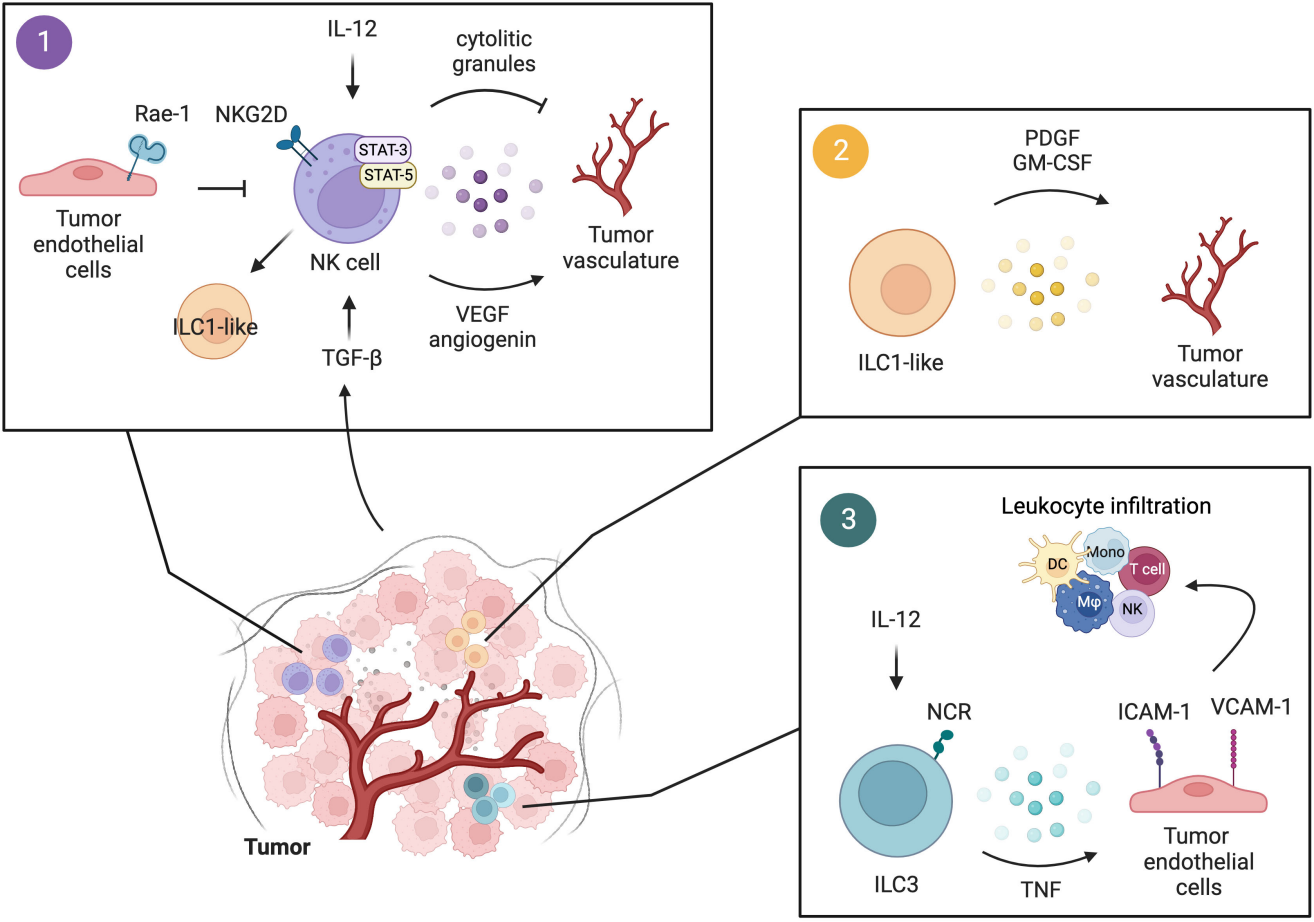
Interactions of innate lymphocytes with the tumor vasculature. 1) Upon IL-12 stimulation, perforin and granzymes released by NK cells act on the tumor vasculature and suppress tumor angiogenesis. Tumor-infiltrating NK cells modulate tumor angiogenesis also through the secretion of VEGF and angiogenin in a STAT-3/5-dependent manner. This pro-angiogenic activity of NK cells can be fostered by TGF-β in the TME, which also induces the conversion of NK cells to a more immature phenotype of ILC1-like cells. Tumor endothelial cells expressing Rae-1 induce constitutive activation of NKG2D^+^ NK cells leading to reduced NKG2D-dependent responses in NK cells and thus impaired anti-tumor functions. 2) The presence of TGF-β in the TME promotes NK cell conversion to an ILC1-like phenotype, characterized by diminished cytotoxic activity and increased release of pro-angiogenic factors, namely PDGF and GM-CSF. 3) Human and murine NCR^+^ ILC3 by secreting pro-inflammatory mediators, including TNF, induce the expression of ICAM-1 and VCAM-1 on tumor endothelial cells, favoring leukocyte infiltration and the establishment of a pro-inflammatory environment. Figure generated with BioRender.

ILC privileged position within tissues allows them to communicate and regulate the homeostatic functions of structural cells (103), including endothelial cells (104). In the next session, we will focus on the pathways regulating the crosstalk between innate lymphocytes and endothelial cells in cancer (**Figure 2**).

### 3.1 NK cells and tumor angiogenesis

NK cells were initially shown to exert anti-angiogenic functions. In a murine model of Burkitt lymphoma, it was reported that, upon IL-12 treatment, perforin- and granzyme-positive tumor-infiltrating NK cells accumulated in tumor tissues and located close to the vessels (105). This cytotoxic NK cell infiltration correlated with reduced new vessel formation (105). However, more recent evidence reported that NK cells exerted pro-angiogenic functions in humans. Around 90% of human circulating NK cells show a CD56^dim^ phenotype and are characterized by cytotoxic functions, whereas 10% is composed of CD56^bright^ cells, mainly showing immunoregulatory functions (106, 107). Although genetically different, this CD56^bright^ NK cell population shared many similarities with a unique subset of CD56^superbright^CD16^-^ decidual NK cells (dNK) deprived of cytotoxic activity, but involved in the modulation of decidual vascular growth during pregnancy through the secretion of angiogenic factors, such as VEGF, PlGF, and IL-8 (108). When transplanted together with human choriocarcinoma-like JEG-3 tumor cells in the subcutis of nude mice, dNK cells markedly supported VEGF and PlGF-dependent tumor vascularization (108). Furthermore, a subset of NK cells resembling dNK cell features was identified in human CRC. These dNK-like cells exhibited increased production of pro-angiogenic factors, such as angiogenin and VEGF, as well as pro-invasive factors, including MMP-9, TIMP-1, and TIMP-2. Of note, the dNK-like cell production of angiogenin and VEGF was dependent on STAT-3 and STAT-5 activation, whereas the production of MMP-9, TIMP-1, and TIMP-2 was STAT-3/5-independent (109). Conversely, Gotthard and colleagues showed that in a model of murine lymphoma (26), STAT-5-deficient NK cells expressed elevated levels of VEGF and were able to induce endothelial cell sprouting *ex vivo*. Selective deletion of VEGF in NKp46^+^ innate cells, including NK cells, showed reduced tumor burden correlated with reduced tumor vascularization (110). Therefore, depending on the host species and the origin of the tumor, STAT-5 could have opposite roles in regulating NK cell pro-angiogenic functions. Compared to CD56^+^CD16^+^ NK cells, human CD56^+^CD16^-^ NK cells produce higher levels of pro-angiogenic factors, namely VEGF, IL-8, and PlGF (111). Interestingly, it was shown that such production was enhanced in CD56^+^CD16^-^ NK cells isolated from squamous cell carcinoma patients and was TGF-β-dependent (111). This suggested a role for TGF-β in modulating NK cell pro-angiogenic functions. TGF-β is a cytokine exerting immunosuppressive functions in the TME (112) and it was shown to modulate the expression of CD16 and thus the NK cell cytotoxic potential (113). In accordance with this, TGF-β in the TME of MCA-induced fibrosarcoma promoted NK cell conversion into a more immature and less cytotoxic phenotype, defined as ILC1-like, characterized by a pro-angiogenic signature (114).

NKG2D is an activating receptor expressed by NK cells that has an important role in NK cell-dependent tumor immunity, including resistance to metastasis (115). Recent work from Thompson and colleagues showed that Rae-1, one of the ligands for NKG2D, was expressed by certain populations of endothelial cells at steady state (116). Rae-1 was detectable in endothelial cells from lymph nodes and spleen but was not detected in endothelial cells from highly vascularized tissues, such as the liver, lung, and heart (116). The constitutive engagement of NK cell NKG2D by endothelial cell Rae-1 led to NKG2D downregulation and diminished NK cell responses (116). Interestingly, Rae-1 expression was induced on endothelial cells from primary melanoma (B16) and correlated with reduced NK cell anti-tumor functions (116). Rae-1 expression was mainly found in endothelial cells of High-Endothelial Venules (HEV), which are blood vessels responsible for lymphocyte migration to lymph nodes (and tumors) (117, 118). These data not only suggest that endothelial cells could control NK cell anti-tumor functions in primary tumors but eventually also at metastatic sites, since NK cell trafficking to the lymph nodes has an important role in NK cell-dependent resistance to metastasis (119).

Taken together, these observations suggest that depending on the host species, the tissue of origin and the cytokine signature of the TME, NK cells can be manipulated to acquire a pro-tumoral and pro-angiogenic phenotype. However, further investigations are needed to better elucidate the molecular pathways responsible for the acquisition of such a pro-angiogenic phenotype in tumor-infiltrating NK cells.

### 3.2 Helper-like ILC and endothelial cell activation

In recent years, the contribution of ILC to tumor immunity was broadly reported (99, 120, 121). As shown for macrophages, depending on the characteristics of the TME, ILC can acquire diverse activation profiles and can switch from an anti-tumor to a pro-tumor phenotype (99, 122). As potent cytokine producers, ILC can crosstalk with other immune and non-immune cells in the TME, including endothelial cells. In a murine model of melanoma overexpressing IL-12 (B16-IL-12), NKp46^+^ ILC3 were shown to infiltrate the tumor and mediate the upregulation of ICAM-1 and VCAM-1 adhesion molecules on tumor vessels leading to the generation of a pro-inflammatory microvascular environment (123). This promoted leukocyte recruitment to the tumor bed and suppression of tumor growth (123). Human NCR^+^ ILC3 were shown to accumulate in human non-small cell lung cancer (NSCLC) where they represented an important source of innate cytokines, including TNF and IL-8 (124). In accordance with the work of Eisering and colleagues, tumor-infiltrating human NCR^+^ ILC3-derived cytokines promoted the adhesion of *in vitro* cultured endothelial cells and upregulated their expression of ICAM-1 and VCAM-1 (124). This suggested that both murine and human NCR^+^ ILC3 could modulate tumor endothelial cell activation, promote leukocyte recruitment, favor the establishment of a pro-inflammatory microenvironment, and thus exert anti-tumoral functions.

A study conducted by Shikhagaie and colleagues identified a novel neuropilin 1 (NRP-1)^+^ ILC3 subset that is endowed with angiogenic potential (125). NRP-1 is a multifunctional receptor involved in the signal transduction of various angiogenic proteins, including VEGF (126). During pulmonary pathogenesis in chronic obstructive pulmonary disease (COPD), human NRP-1^+^ ILC3 located in proximity to blood vessels and lung ectopic lymphoid tissues, where they induced VCAM-1 and ICAM-1 expression on mesenchymal stromal cells, and efficiently responded to VEGF-A (125). However, the study did not provide any clear evidence of a direct crosstalk between the NRP-1^+^ ILC3 and the endothelial compartment. Further studies are therefore warranted to better understand the role of NRP1^+^ ILC3 in endothelial cell activation and to eventually extend this to cancer.

Since the early 2000s, IL-17 and endothelial cell crosstalk was evaluated in homeostasis and disease, including cancer (127–131). In primary human lung carcinomas, IL-17 was linked to the cancer cell production of a variety of pro-angiogenic factors, such as IL-6, IL-8, and VEGF (132), as well as to the upregulation of VEGF-C, a growth factor involved in lymphangiogenesis (133). Furthermore, it was proposed that murine pulmonary metastatic progression could be facilitated through the IL-17-dependent induction of VCAM-1 expression on lung endothelial cells, which correlated with altered vessel permeability and enhanced transendothelial migration of tumor cells (134). IL-17 was also recognized for its pro-inflammatory role in the development of intestinal malignancies (135). In colorectal and gastric cancers, IL-17 was described to interact with tumor cells to produce angiogenic mediators, including VEGF (129, 136). CRC-infiltrating Th17 cells induced the expression of G-CSF by CAF leading to the recruitment to the TME of CD11b^+^Gr1^+^ myeloid cells endowed with pro-angiogenic features, and thus promoting resistance to VEGF blockade therapy (137). In some of those studies, the cellular source of IL-17 was identified and mainly comprised of Th17 cells. However, ILC3 are also important producers of IL-17 (100) and ILC3-derived IL-17 was correlated with cancer progression (138). Therefore, further studies are required to elucidate the potential contribution of IL-17-producing ILC3 to tumor angiogenesis.

IL-22 is a cytokine belonging to the IL-10 family produced by ILC3 to maintain tissue homeostasis (139). The role of IL-22 in cancer is complex since IL-22 was shown to either promote or restrain tumor progression (139). After binding to its receptor (IL-22RA1), which is selectively expressed by non-hematopoietic cells (140), IL-22 induces phosphorylation of STAT-3. STAT-3 is a pleiotropic transcription factor that upon constitutive activation becomes oncogenic (141). High levels of IL-22 were detected in colonic cancer tissues, where IL-22 exerted pro-tumoral functions by promoting cancer cell activation of STAT-3, reducing tumor cell apoptosis, and increasing the levels of VEGF in the TME (142). Importantly, emerging evidence showed that also endothelial cells can express IL-22RA1 and thus can respond to IL-22 (143). Upon IL-22 stimulation, STAT-3 activation led to enhanced endothelial cell proliferation and survival, and promoted the growth of murine T-cell lymphoma (EL4) (143). By regulating the expression of the pro-invasive molecule alanyl aminopeptidase (Anpep) on the surface of endothelial cells, hepatic invariant-NKT (iNKT)-derived IL-22 modulated endothelial cell permeability and favored the seeding of circulating cancer cells in the liver, promoting CRC liver metastases (144). However, the molecular mechanisms by which IL-22 regulates endothelial cell permeability at metastatic sites remain unclear. These findings suggest a role for IL-22 to directly or indirectly shape endothelial cell functions in cancer. Although IL-22^+^ ILC3 were shown to have an important role in cancer (145, 146), the contribution of ILC3-derived IL-22 to tumor angiogenesis has not been elucidated yet.

ILC3 emerge as potential players in the regulation of tumor vasculature but the contribution of ILC1 and ILC2 to tumor angiogenesis is less understood. NK cell plasticity towards ILC1 was suggested as a strategy employed by tumors to evade immune surveillance (114). However, little is known about the potential of helper-like ILC1 to regulate blood vessel formation in cancer. This might be related to the reduced panel of unique markers that can be used to properly discern between NK cells and ILC1, particularly in cancer, where immune cells acquire mixed phenotypes and show high levels of heterogeneity. When it comes to ILC2, recent evidence showed that murine ILC2 could produce VEGF-A in asthmatic inflammation (147), but there is no evidence that this ILC2 pro-angiogenic function could be observed in cancer. Therefore, further investigations are needed to understand whether ILC1 and ILC2 could modulate cancer angiogenesis.

## 4 Conclusions and perspectives

**Figure 3:**
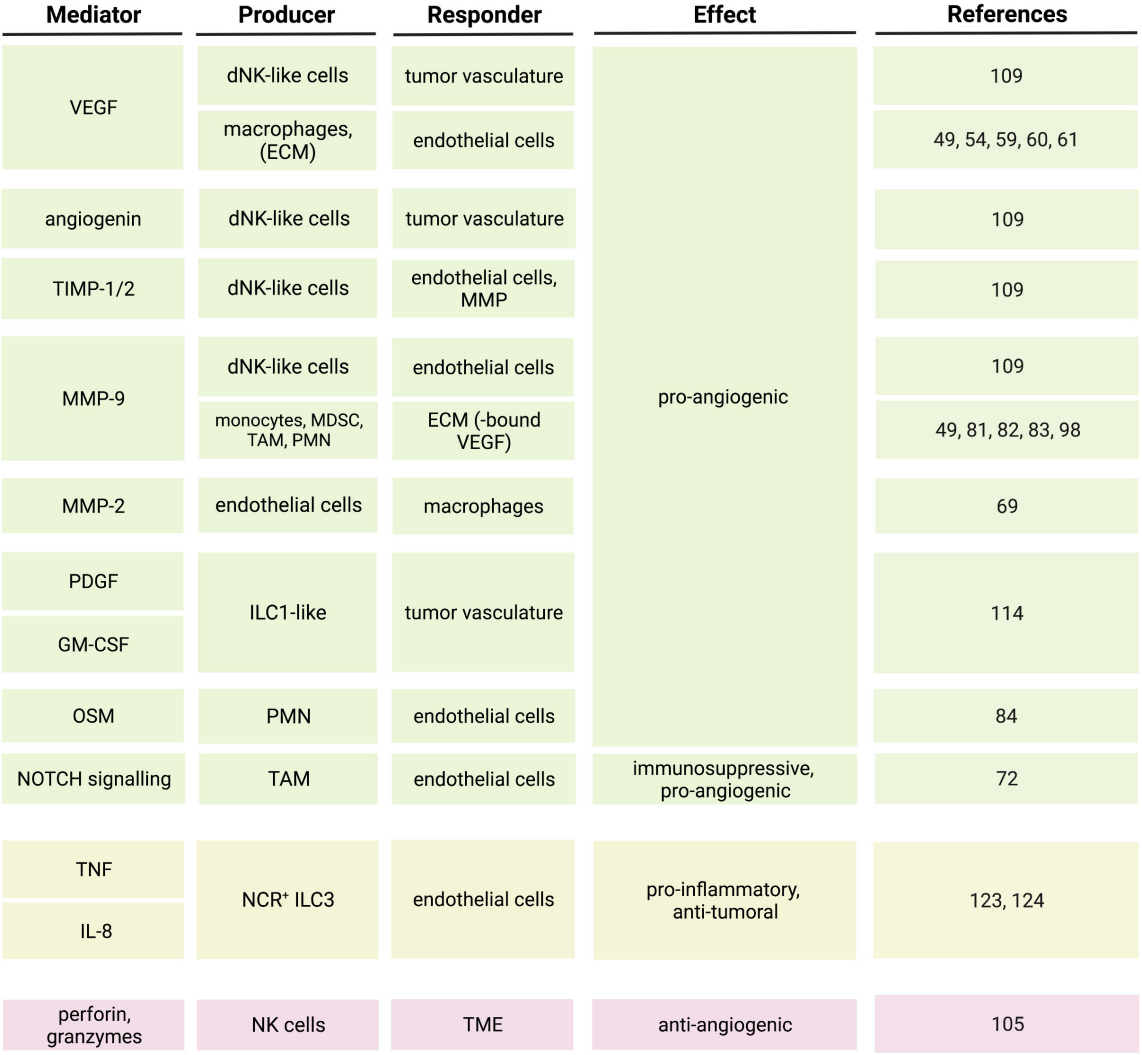
Soluble mediators and pathways regulating the crosstalk between innate immune cells and endothelial cells in the tumor microenvironment.

In this review, we reported evidence of the crosstalk between endothelial cells and both myeloid cells and innate lymphocytes in the TME and the pathways that until now were described to modulate such a crosstalk. VEGF represents one of the major regulators of the interaction between myeloid cells and endothelial cells in cancer (44–46, 81), but is also involved in the crosstalk with NK cells (108–111), and the interaction between ILC and tumor endothelial cells is regulated by TNF, IL-8 and TGF-β (114, 124) (**Figure 3**). However, other molecules, including angiopoietins and metalloproteinases, also influence the crosstalk between endothelial cells and innate immune cells in the TME (46–48, 74, 76, 77, 81, 109) (**Figure 3**). The interaction between innate immune cells and endothelial cells in cancer is complex and sometimes ambiguous, since, depending on the cytokine signature of the TME, it can lead to either tumor regression or progression. Therefore, targeting the pro-angiogenic mediators in the TME with the aim to normalize the tumor vasculature and thus restraining the neoplastic growth might not have an obvious outcome. One of the therapeutic approaches aiming at interfering with tumor angiogenesis consists of anti-VEGF treatment. Anti-VEGF treatment leads to decreased vessel diameter and density, suggesting for a normalization of the tumor vasculature, and increased uptake of chemotherapy agents (148). However, the blockade of VEGF could also impact the migration and function of VEGF responsive immune cells (55, 56, 125, 149) and their progenitors (150), potentially interfering with cancer immune surveillance. Furthermore, clinical data indicated that anti-angiogenic treatments are not always efficient in normalizing the tumor vasculature and could lead to therapy resistance due to the presence of compensatory angiogenic pathways (151). Therefore, a combination approach should be taken into consideration when it comes to anti-angiogenic therapies. A correlation between the angiogenic profile and the response to immune-checkpoint blockade (ICB) was reported (118, 152, 153) and anti-angiogenic treatments were shown to enhance the efficacy of immunotherapies (154–156). Combined treatments with anti-angiogenic agents (anti-VEGF or anti-VEGFR2) and ICB stimulated vessel normalization and the formation of HEV, favoring immune control of tumor progression (154, 155), and induced anti-tumor T cell responses (156). In addition, it was shown that radiotherapy could further enhance the anti-cancer effect of combined anti-angiogenic and ICB treatments (157). These evidences strengthen the concept that indeed the crosstalk between tumor immunity and tumor vasculature has an important role in cancer progression and could be efficiently targeted. Recent studies indicated a contribution of tumor-infiltrating innate lymphocytes in determining the efficacy of ICB (121, 158–160). Importantly, the same stimulator of interferon genes (STING) that was shown to be involved in the suppression of tumor angiogenesis and vessel normalization (161) also contributed to tumor monocyte reprogramming and NK cell-mediated anti-tumor immunity (162). The activation of the STING/IFN I axis in tumors not only improved the innate cell-dependent efficacy of ICB (162), but was also shown to enhance the efficacy of anti-angiogenic therapies, namely VEGFR2 blockade (161). This suggests that combined therapies targeting pro-angiogenic factors and unleashing innate-dependent anti-tumor immunity could not only have therapeutic effects by their selves but could also improve the efficacy of other immunotherapies. Further investigations may extend our understanding of the mechanisms by which endothelial cells crosstalk with myeloid and innate lymphoid cells. Elucidation of the pathways regulating interactions between innate immune cells and the tumor vasculature will allow for a better design of those potential combined therapies that could interfere with tumor angiogenesis, promote normalization of tumor vessels and immune cell trafficking and activation, and enhance the efficacy of immunotherapies.

## Author contributions

All authors contributed to the article and approved the submitted version.
